# Sleep Quality and Depressive Symptoms in Clinically Stable Pediatric Familial Mediterranean Fever: Associations with Disease Severity and Developmental Factors

**DOI:** 10.3390/children13070950

**Published:** 2026-07-20

**Authors:** Begum Baris Cetinkaya, Fatih Battal

**Affiliations:** 1 Department of Pediatrics, Faculty of Medicine, Bandirma Onyedi Eylul University, Balikesir 10200, Türkiye; bcetinkaya@bandirma.edu.tr; 2 Department of Pediatrics, Faculty of Medicine, Canakkale Onsekiz Mart University, Canakkale 17100, Türkiye

**Keywords:** child, depression, familial mediterranean fever, psychosocial functioning, sleep quality

## Abstract

**Highlights:**

**What are the main findings?**

- Clinically stable children and adolescents with FMF did not exhibit poorer sleep quality or greater depressive symptom severity than age- and sex-comparable healthy controls.
- Increasing age, particularly adolescence, was independently associated with poor sleep quality, whereas disease severity assessed using the International Severity Scoring System for Familial Mediterranean Fever (ISSF) was not.

**What are the implications of the main findings?**

- Developmental factors may contribute more strongly to sleep quality than clinically assessed disease severity in children and adolescents with FMF.
- Routine assessment of sleep quality and emotional well-being, particularly during adolescence, should be considered as part of comprehensive multidisciplinary care for pediatric FMF.

**Abstract:**

**Background:** Familial Mediterranean Fever (FMF) is a chronic autoinflammatory disease that may adversely affect sleep quality, emotional well-being, and other patient-reported outcomes during childhood and adolescence. However, previous pediatric studies have frequently included heterogeneous patient populations with varying disease activity, limiting understanding of the relationship between disease severity and psychosocial functioning. **Objective:** To evaluate sleep quality and depressive symptoms in clinically stable children and adolescents with FMF during attack-free periods and to investigate their relationships with disease severity assessed using the International Severity Scoring System for Familial Mediterranean Fever (ISSF) and developmental factors. **Methods:** This cross-sectional case–control study included 72 children and adolescents with FMF and 88 age- and sex-comparable healthy controls. All patients with FMF were evaluated during clinically stable attack-free periods while receiving maintenance colchicine therapy. Depressive symptoms and sleep quality were assessed using the Children’s Depression Inventory (CDI) and the Pittsburgh Sleep Quality Index (PSQI), respectively. Disease severity was evaluated using the ISSF. Multivariable logistic regression analysis was performed to identify factors independently associated with poor sleep quality. **Results:** Healthy controls demonstrated significantly higher mean CDI and PSQI scores than patients with FMF (CDI: 10.3 ± 6.8 vs. 8.2 ± 5.3, *p* = 0.048; PSQI: 4.7 ± 2.7 vs. 3.0 ± 2.3, *p* < 0.001). Poor sleep quality (PSQI ≥ 5) was more frequent among controls (46.6% vs. 25.0%, *p* = 0.006). Within the FMF cohort, adolescents (13–18 years) had significantly poorer sleep quality than younger children (*p* = 0.005). Increasing age was independently associated with poor sleep quality (OR 1.24, 95% CI 1.07–1.43; *p* = 0.003), whereas FMF diagnosis was not independently associated with poor sleep quality after adjustment for age and sex. No significant association was identified between ISSF-defined disease severity and psychosocial outcomes. Sleep quality showed a modest positive correlation with depressive symptom severity (Spearman’s ρ = 0.313, *p* = 0.007). **Conclusions:** Children and adolescents with FMF receiving maintenance colchicine therapy during clinically stable attack-free periods did not demonstrate poorer sleep quality or greater depressive symptom severity than age- and sex-comparable healthy controls. Increasing age appeared to be more closely associated with poor sleep quality than clinically assessed disease severity, whereas sleep quality showed a modest association with depressive symptom severity. These findings suggest that incorporating routine assessment of sleep quality and psychological well-being into multidisciplinary follow-up may facilitate a more comprehensive evaluation of children and adolescents with FMF.

## 1. Introduction

Familial Mediterranean fever (FMF) is the most common monogenic autoinflammatory disease, resulting from dysregulation of the innate immune system and characterized by recurrent episodes of fever and sterile serositis [1]. The disease typically begins during childhood and is particularly prevalent among populations originating from the Mediterranean basin, the Middle East, and North Africa, especially Turks, Armenians, Arabs, and Sephardic Jews [2]. Owing to its early onset and lifelong course, FMF extends beyond recurrent inflammatory attacks and may affect multiple aspects of child health, including emotional development, sleep regulation, school functioning, social participation, and psychosocial adaptation throughout childhood and adolescence. Accordingly, contemporary management of pediatric FMF increasingly incorporates measures of patient well-being alongside conventional assessments of inflammatory disease activity.

The molecular basis of FMF involves gain-of-function mutations in the *MEFV* gene located on the short arm of chromosome 16, which encodes pyrin, a key regulator of inflammasome activation and interleukin-1β (IL-1β) production. Pyrin dysfunction results in excessive activation of the innate immune response, leading to recurrent, self-limited episodes of sterile inflammation involving the peritoneum, pleura, and joints [3]. More than 400 MEFV variants have been identified, with exon 10 mutations—particularly M694V—being consistently associated with earlier disease onset, greater disease severity, colchicine resistance, and an increased risk of amyloidosis [4,5,6,7]. Although heterozygous variants are generally associated with milder phenotypes, genotype alone does not fully explain the marked variability in clinical manifestations, suggesting that additional immunological, environmental, and developmental factors contribute to disease expression [8,9]. Emerging evidence indicates that the biological consequences of persistent innate immune activation extend beyond peripheral inflammation. Beyond its role in systemic inflammation, chronic innate immune activation has been implicated in neuroimmune signaling pathways involved in sleep regulation, emotional processing, and mood disorders, providing a potential biological link between inflammatory disease activity and behavioral and emotional health. This neuroimmune perspective provides a mechanistic rationale for investigating sleep quality and depressive symptoms as clinically relevant outcomes in children with FMF.

FMF is a lifelong disease requiring continuous treatment and regular clinical follow-up [10]. Colchicine remains the cornerstone of therapy, effectively reducing attack frequency and severity, preventing amyloidosis, and improving long-term clinical outcomes and health-related quality of life (QoL) [11]. Nevertheless, approximately 5–10% of patients exhibit colchicine-resistant disease despite receiving the maximum tolerated dose and may require biologic therapies targeting interleukin-1 (IL-1) [12]. Although contemporary treatment strategies have substantially improved inflammatory disease control and prognosis, disease control alone may not fully capture the overall burden experienced by affected children and adolescents. Many children continue to experience psychosocial challenges that cannot be fully explained by inflammatory activity alone, highlighting the importance of evaluating outcomes beyond conventional clinical measures. Accordingly, comprehensive assessment of pediatric FMF now extends beyond inflammatory control to include the child’s daily functioning and perceived well-being.

Although recurrent inflammatory attacks represent the hallmark of FMF, accumulating evidence indicates that the disease imposes a substantially broader burden encompassing physical, psychological, developmental, and social domains. Previous studies have consistently demonstrated impaired health-related quality of life (QoL) in both adult and pediatric patients, with frequent attacks, persistent disease activity, and greater disease severity being associated with poorer physical, emotional, and social functioning [13,14,15,16]. These findings indicate that the impact of FMF extends well beyond inflammatory manifestations and should therefore be viewed within a broader biopsychosocial framework that extends beyond conventional clinical measures.

Beyond conventional clinical outcomes, recent pediatric studies have identified fatigue as an important contributor to disease burden, demonstrating close associations between fatigue, sleep quality, physical activity, and disease activity in children and adolescents with FMF [13,17,18,19,20]. Furthermore, fatigue has emerged as a clinically relevant patient-reported outcome reflecting the cumulative impact of chronic inflammation on daily functioning and overall well-being [21]. Collectively, these observations suggest that psychosocial functioning in pediatric FMF is multidimensional and cannot be adequately characterized by inflammatory disease activity alone. Because childhood and adolescence represent critical periods of neurodevelopment and social maturation, chronic inflammatory diseases may interfere with emotional regulation, academic performance, peer relationships, and psychosocial adaptation. Consequently, comprehensive assessment increasingly integrates measures of daily functioning together with traditional indicators of disease activity and treatment response. Recent evidence also indicates that health-related quality of life, fatigue, and sleep quality are closely interconnected, supporting a multidimensional approach to disease assessment that extends beyond conventional clinical outcomes [17].

Sleep disturbances have been reported more frequently in children and adolescents with FMF and appear to be associated with disease activity and clinical severity [18]. Sleep plays a fundamental role in neurodevelopment, cognitive performance, emotional regulation, and immune homeostasis throughout childhood. Chronic inflammation may disrupt these processes through neuroimmune mechanisms involving pro-inflammatory cytokines, particularly interleukin-1β (IL-1β), which is implicated in both inflammatory responses and physiological sleep regulation [22,23]. Together, these observations support considering sleep quality as a clinically meaningful outcome rather than merely a secondary consequence of chronic disease.

Moreover, adolescence is characterized by profound developmental changes in circadian rhythm, sleep regulation, and emotional maturation, making this period particularly vulnerable to sleep disturbances and emotional difficulties. Consequently, the interaction between chronic inflammatory disease and normal developmental processes may substantially influence sleep-related and emotional outcomes in pediatric FMF.

Most previous studies have interpreted psychological functioning in pediatric FMF primarily in relation to inflammatory activity and disease severity. However, research on pediatric chronic illnesses suggests that emotional adjustment is shaped by a broader range of factors, including resilience, coping strategies, family functioning, and family-centered care. Consequently, favorable psychosocial adaptation may occur despite chronic illness when disease control is optimized and adequate family support is available. Rather than contradicting previous reports of impaired sleep quality, fatigue, anxiety, depression, and reduced quality of life in pediatric FMF [13,15,17,18,19,20,24,25], this perspective suggests that these manifestations reflect the combined influence of inflammation, development, and environmental context. To provide an overview of the existing evidence, the principal pediatric studies evaluating sleep quality, depressive symptoms, fatigue, and related psychosocial outcomes in children and adolescents with FMF are summarized in Table 1.

Despite accumulating evidence demonstrating impaired sleep quality, fatigue, anxiety, depressive symptoms, and reduced health-related quality of life in pediatric FMF [13,15,17,18,19,20,24,25], several important knowledge gaps remain. Previous studies have generally evaluated heterogeneous patient populations with varying disease activity, have often included patients during both attack and attack-free periods, and have rarely examined psychosocial outcomes in relation to standardized measures of disease severity. Furthermore, relatively little is known about the extent to which sleep quality and depressive symptoms are influenced by clinically assessed disease severity, developmental stage, or broader psychosocial factors in children with clinically stable FMF receiving continuous colchicine therapy. Clarifying these relationships is clinically relevant because distinguishing the effects of inflammatory disease burden from those of normal developmental and psychosocial processes may improve the interpretation of patient-reported outcomes and facilitate more individualized long-term care.

**Figure 1:**
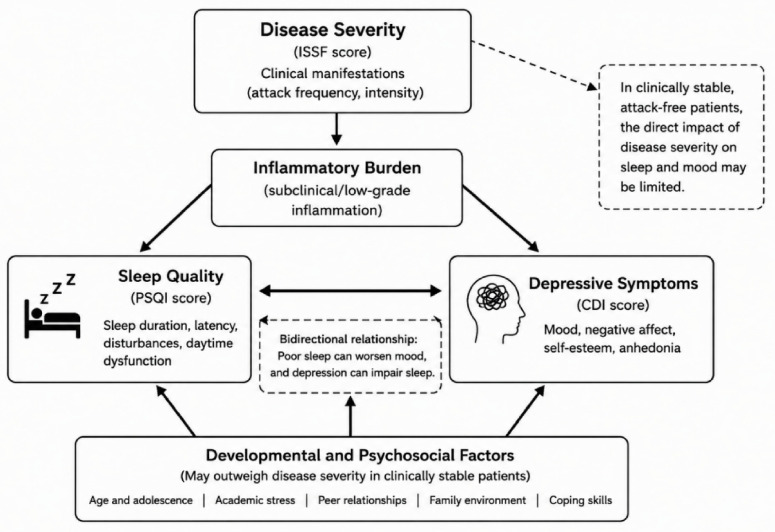
Proposed conceptual framework illustrating the hypothesized relationships among inflammatory burden, disease severity, developmental factors, sleep quality, and depressive symptoms in clinically stable pediatric Familial Mediterranean Fever.

Based on these identified knowledge gaps, we developed a conceptual framework illustrating the hypothesized relationships among inflammatory burden, disease severity, developmental factors, sleep quality, and depressive symptoms in clinically stable pediatric FMF (Figure 1).

Based on these considerations, the present study aimed to evaluate sleep quality and depressive symptoms in children and adolescents with FMF receiving continuous colchicine therapy during clinically stable attack-free periods. In addition, we investigated whether psychosocial outcomes were associated with disease severity assessed using the International Severity Scoring System for FMF (ISSF), developmental stage, and selected clinical characteristics. We also examined the association between sleep quality and depressive symptoms and compared these measures with those of healthy controls.

We hypothesized that poorer sleep quality and greater depressive symptom severity would be associated with increasing disease severity and that sleep quality would correlate with depressive symptoms. By minimizing the influence of acute inflammatory attacks through evaluation during clinically stable attack-free periods, the study was designed to better distinguish disease-related effects from developmental and psychosocial influences on patient-reported outcomes.

## 2. Materials and Methods

This cross-sectional case–control study was conducted between October 2018 and May 2019 at the General Pediatrics and Well-Child Clinics of the Department of Pediatrics, Çanakkale Onsekiz Mart University Faculty of Medicine Hospital, Türkiye. The study protocol was developed before participant recruitment and was designed, conducted, and reported in accordance with the Strengthening the Reporting of Observational Studies in Epidemiology (STROBE) statement for observational studies.

### 2.1. Study Design and Participants

The study included children and adolescents aged 6–18 years who were diagnosed with FMF according to the Turkish pediatric FMF diagnostic criteria proposed by Yalçınkaya et al. [5]. Only consecutive eligible patients attending routine outpatient follow-up visits during the study period were invited to participate, thereby minimizing selection bias. Eligible patients had been receiving regular colchicine therapy with documented treatment adherence for at least six months before enrollment.

All clinical and psychological assessments were performed during clinically stable attack-free periods. An attack-free period was defined as the absence of fever, serositis, musculoskeletal manifestations, or other FMF-related symptoms for at least three weeks before study assessment, together with normalization of acute-phase reactants, including C-reactive protein and erythrocyte sedimentation rate [6]. Assessments were intentionally performed during clinically stable periods to minimize the potential confounding effects of acute inflammatory episodes on sleep quality and depressive symptoms. Medication adherence was assessed using parental reports and outpatient follow-up records. Patients missing fewer than three colchicine doses during the preceding six months were considered adherent.

### 2.2. Inclusion and Exclusion Criteria

Eligible participants in the FMF group were children and adolescents aged 6–18 years who had been receiving regular colchicine therapy with documented treatment adherence for at least six months and were clinically stable at the time of assessment. To minimize potential confounding factors affecting sleep quality and depressive symptoms, patients were excluded if they were experiencing an acute FMF attack during evaluation; had another chronic inflammatory, neurological, endocrine, or systemic disease; had a previously diagnosed psychiatric disorder; were under follow-up by child and adolescent psychiatry services for depression, anxiety, behavioral disorders, or sleep-related problems; were receiving psychotropic medication; or were unable to complete the study questionnaires reliably. Children with acute infections within the preceding two weeks were also excluded.

### 2.3. Control Group

The control group consisted of healthy children and adolescents who were age- and sex-comparable to the FMF group and were consecutively recruited from the well-child outpatient clinics during the same study period. They attended the clinics for routine pediatric evaluations, growth monitoring, vaccination follow-up, or pre-participation sports assessments. Children with acute illness, chronic disease, recent infection, sleep-related complaints, psychiatric symptoms, previous psychiatric consultation, ongoing medication use, or any condition potentially affecting sleep or psychological well-being were excluded. These eligibility criteria were applied to minimize potential confounding factors that could independently influence sleep quality and depressive symptoms.

Eligibility was confirmed through a detailed medical history obtained from both children and their parents, physical examination, and review of outpatient medical records. None of the control participants had a history of chronic medical illness or recurrent pain syndromes. Furthermore, none had diagnosed psychiatric disorders, depression, anxiety disorders, sleep disorders, or regular medication use. Information regarding screen time, physical activity, sleep hygiene, academic stress, socioeconomic status, and family-related factors was not systematically collected because these variables were not included in the original study protocol. Accordingly, residual confounding related to these factors cannot be excluded and should be considered when interpreting comparisons between the FMF and control groups. No participant fulfilled the diagnostic criteria for FMF or had a family history suggestive of an autoinflammatory disease.

### 2.4. Sociodemographic and Clinical Data Collection

A structured case report form was completed for all participants at the time of enrollment. Sociodemographic variables included age, sex, height, weight, place of residence, and previous psychiatric consultation history. For patients with FMF, additional disease-related information was extracted from medical records, including age at symptom onset, age at diagnosis, duration of colchicine treatment, *MEFV* mutation profile, medication adherence, and FMF-related complications.

### 2.5. Psychological Assessment

Psychological assessment was performed using standardized and validated self-report screening instruments administered during face-to-face outpatient visits in a quiet clinical setting. The questionnaires were used as screening tools to assess depressive symptoms and sleep quality rather than as diagnostic psychiatric instruments. No structured psychiatric interviews were performed, and no psychiatric diagnoses were established within the scope of the study.

All questionnaires were completed by the participants themselves under the supervision of a pediatrician. For younger children, investigators provided clarification of questionnaire items when necessary to ensure comprehension while avoiding any influence on participants’ responses. Parents were not permitted to answer questionnaire items on behalf of their children. Questionnaire administration followed the same standardized procedure for both the FMF and control groups.

### 2.6. Children’s Depression Inventory (CDI)

Depressive symptoms were assessed using the Children’s Depression Inventory (CDI), developed by Kovacs and validated in Turkish by Oy [26,27]. The CDI is a 27-item self-report questionnaire designed to evaluate depressive symptoms in children and adolescents. Each item is scored from 0 to 2, yielding a total score ranging from 0 to 54. Higher scores indicate greater depressive symptom severity. A total score of ≥19 was considered indicative of clinically significant depressive symptoms.

### 2.7. Pittsburgh Sleep Quality Index (PSQI)

Sleep quality was evaluated using the Pittsburgh Sleep Quality Index (PSQI) [28]. The Turkish version of the PSQI was validated by Ağargün et al. [29]. The questionnaire evaluates subjective sleep quality during the preceding month across seven sleep domains, generating a global score ranging from 0 to 21, with higher scores indicating poorer sleep quality. A global PSQI score of ≥5 was considered indicative of poor sleep quality.

Although the PSQI was originally developed for adults, it has subsequently demonstrated acceptable validity and reliability in pediatric populations and has been successfully applied in studies involving school-aged children and adolescents [30,31]. In the present study, younger children received investigator-assisted clarification of questionnaire wording when necessary to ensure comprehension; however, investigators did not interpret items or influence participants’ responses, and all responses reflected the children’s own answers.

### 2.8. Disease Severity Assessment

Disease severity was assessed using the ISSF [25], a validated clinical instrument developed to provide a standardized assessment of disease severity in patients with FMF. The ISSF consists of nine clinical parameters and classifies disease severity as mild (0–2 points), moderate (3–5 points), or severe (≥6 points). All ISSF scores were calculated at the time of study enrollment using contemporaneous clinical findings and medical records.

### 2.9. Statistical Analysis

Statistical analyses were performed using IBM SPSS Statistics for Windows, version 22.0 (IBM Corp., Armonk, NY, USA). Continuous variables were summarized as mean ± standard deviation (SD) or median (interquartile range [IQR]), as appropriate, whereas categorical variables were presented as frequencies and percentages.

The distribution of continuous variables was assessed using the Kolmogorov–Smirnov and Shapiro–Wilk tests. Because age, Children’s Depression Inventory (CDI) scores, and Pittsburgh Sleep Quality Index (PSQI) scores were not normally distributed, nonparametric statistical methods were used. Between-group comparisons were performed using the Mann–Whitney U test for continuous variables and the chi-square test or Fisher’s exact test for categorical variables, as appropriate. Comparisons among three or more groups were performed using the Kruskal–Wallis test.

Correlations between disease severity assessed by the ISSF, depressive symptoms (CDI), and sleep quality (PSQI) were evaluated using Spearman’s rank correlation coefficient. Age-stratified subgroup analyses were additionally performed for children (6–12 years) and adolescents (13–18 years) to explore potential developmental differences in psychosocial outcomes.

Multivariable logistic regression analysis was performed to identify factors independently associated with poor sleep quality (PSQI ≥ 5). Poor sleep quality was entered as the dependent variable, whereas clinically relevant variables (FMF diagnosis, age, and sex) were entered simultaneously as independent variables. Adjusted odds ratios (ORs) and corresponding 95% confidence intervals (CIs) were calculated. Because ISSF scores were available only for participants with FMF and only five patients were classified as having severe disease, ISSF was not included in the multivariable model to avoid overfitting and unstable parameter estimates. Multicollinearity among independent variables was assessed before model construction.

No formal a priori sample size calculation was performed because this was an exploratory observational study in which all consecutive eligible participants presenting during the predefined study period were included. Participants with incomplete questionnaire data were excluded before statistical analysis; therefore, no imputation for missing data was performed. All statistical tests were two-sided, and a *p*-value < 0.05 was considered statistically significant.

### 2.10. Ethical Considerations

Written informed consent was obtained from the parents or legal guardians of all participants before enrollment. The study protocol was approved by the Ethics Committee of Çanakkale Onsekiz Mart University Faculty of Medicine (Protocol No. 18920478-050.04.04, dated 17 October 2018). The study was conducted in accordance with the principles of the Declaration of Helsinki.

## 3. Results

### 3.1. Participant Selection and Study Population

During the study period, 91 consecutive children and adolescents with FMF were assessed for eligibility. Nineteen patients were excluded because they were experiencing an acute FMF attack at the time of evaluation (*n* = 6), had a concomitant chronic systemic disease (*n* = 3), had a diagnosed psychiatric disorder or were receiving psychotropic medication (*n* = 5), or returned incomplete questionnaires (*n* = 5). After application of the predefined eligibility criteria, 72 clinically stable children and adolescents with FMF were included in the final analysis.

For the control group, 108 consecutive healthy children and adolescents attending the well-child outpatient clinics were screened for eligibility. Twenty participants were excluded because of acute illness (*n* = 6), sleep-related complaints (*n* = 4), psychiatric history (*n* = 3), ongoing medication use (*n* = 2), or incomplete questionnaires (*n* = 5). After application of the predefined eligibility criteria, 88 age- and sex-comparable healthy participants were included in the final analysis. No eligible participant who fulfilled the inclusion criteria declined study participation.

**Figure 2:**
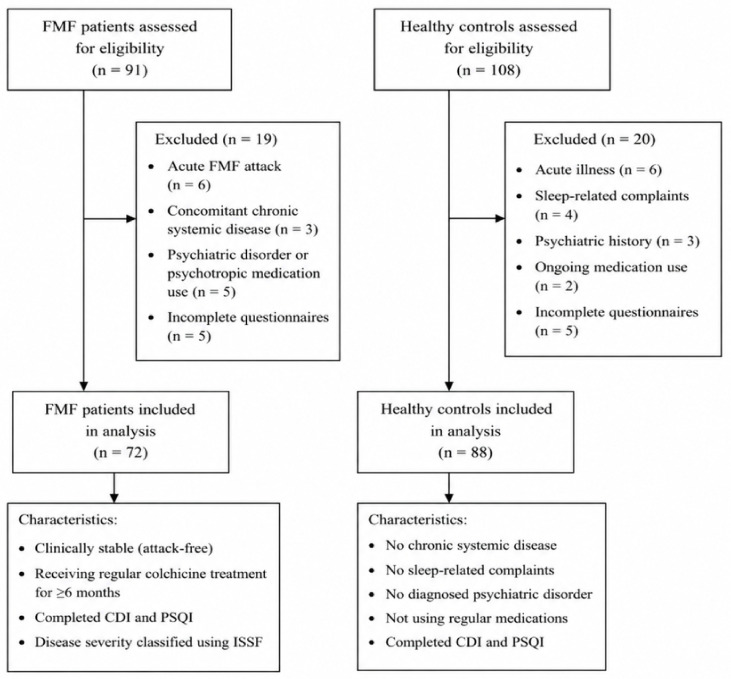
Flow diagram of participant selection and study inclusion according to the predefined eligibility criteria. **Abbreviations: FMF**, Familial Mediterranean Fever; **CDI**, Children’s Depression Inventory; **PSQI**, Pittsburgh Sleep Quality Index; **ISSF**, International Severity Scoring System for Familial Mediterranean Fever.

The final study population therefore consisted of 72 clinically stable children and adolescents with FMF and 88 age- and sex-comparable healthy controls. The participant selection process is illustrated in Figure 2.

### 3.2. Sociodemographic and Clinical Characteristics

The sociodemographic and clinical characteristics of the study population are summarized in Table 2. The FMF and control groups were comparable with respect to age (10.6 ± 3.3 vs. 11.5 ± 3.1 years, *p* = 0.081) and sex distribution (female: 48.6% vs. 50.0%, *p* = 0.861).

Within the FMF cohort, the mean age at symptom onset was 6.2 ± 3.3 years, whereas the mean age at diagnosis and initiation of colchicine therapy was 7.9 ± 3.4 years. Accordingly, the mean interval between symptom onset and diagnosis was approximately 1.7 years. Most patients (86.1%) reported regular colchicine adherence during the preceding six months. Regarding the *MEFV* genotype, compound heterozygous variants were the most common (43.1%), followed by heterozygous (31.9%) and homozygous variants (25.0%).

### 3.3. Comparison of Psychosocial Assessment Scores

The psychosocial assessment results are summarized in Table 3. Compared with the FMF group, healthy controls demonstrated significantly higher mean Children’s Depression Inventory (CDI) scores (10.3 ± 6.8 vs. 8.2 ± 5.3, *p* = 0.048) and Pittsburgh Sleep Quality Index (PSQI) scores (4.7 ± 2.7 vs. 3.0 ± 2.3, *p* < 0.001). Poor sleep quality (PSQI ≥ 5) was also more frequent among healthy controls than among patients with FMF (46.6% vs. 25.0%, *p* = 0.006). In contrast, the prevalence of clinically significant depressive symptoms (CDI ≥ 19) did not differ significantly between the groups (9.1% vs. 5.6%, *p* = 0.411).

### 3.4. Disease Severity According to the International Severity Scoring System

Disease severity according to the ISSF is presented in Table 4. Within the FMF cohort, the mean ISSF score was 2.4 ± 1.2, indicating that the study population predominantly comprised patients with mild disease severity. Of the 72 patients with FMF, 39 (54.2%) were classified as having mild disease, 28 (38.9%) as moderate disease, and 5 (6.9%) as severe disease. Mean Children’s Depression Inventory (CDI) scores increased across ISSF categories, from 7.5 ± 4.9 in patients with mild disease to 11.6 ± 6.1 in those with severe disease; however, the difference did not reach statistical significance (*p* = 0.182). Similarly, mean Pittsburgh Sleep Quality Index (PSQI) scores increased with disease severity (2.8 ± 2.1, 3.2 ± 2.4, and 5.2 ± 2.4 for mild, moderate, and severe disease, respectively), although this trend was also not statistically significant (*p* = 0.146). Poor sleep quality (PSQI ≥ 5) was observed in 25.6% of patients with mild disease, 17.9% of those with moderate disease, and 60.0% of patients with severe disease (*p* = 0.071). Although none of these comparisons reached statistical significance, mean CDI and PSQI scores tended to increase with increasing disease severity. The proportion of patients with poor sleep quality was highest in the severe disease group.

### 3.5. Age-Based Subgroup Analyses

**Figure 3:**
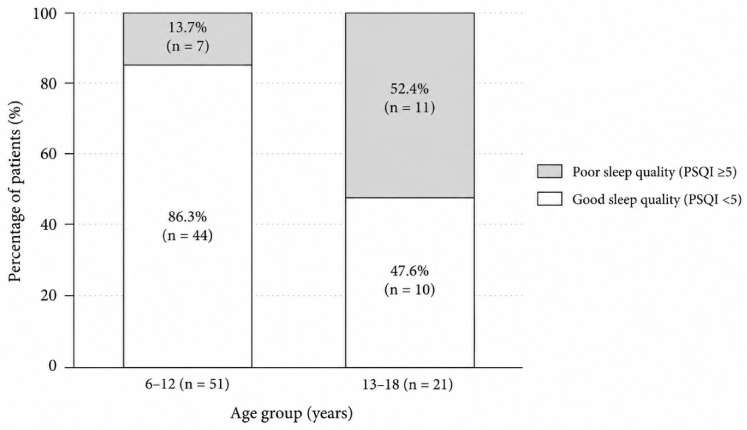
Distribution of sleep quality according to age group in children and adolescents with Familial Mediterranean Fever. The proportions of patients with good sleep quality (PSQI < 5) and poor sleep quality (PSQI ≥ 5) are presented for children aged 6–12 years and adolescents aged 13–18 years.

Age-stratified analyses of the FMF cohort are presented in Table 5 and Figure 3. Adolescents aged 13–18 years had significantly higher Pittsburgh Sleep Quality Index (PSQI) scores than children aged 6–12 years (4.2 ± 2.7 vs. 2.5 ± 2.0, *p* = 0.005). Likewise, poor sleep quality (PSQI ≥ 5) was observed more frequently among adolescents than among younger children (52.4% vs. 13.7%, *p* = 0.005). No statistically significant differences were observed between age groups in Children’s Depression Inventory (CDI) scores (8.9 ± 5.7 vs. 7.9 ± 5.1, *p* = 0.462) or ISSF scores (2.6 ± 1.3 vs. 2.3 ± 1.1, *p* = 0.334).

### 3.6. Genotype-Based Analyses

Genotype-based analyses are presented in Supplementary Table S1. Patients carrying homozygous *MEFV* mutations had significantly higher Children’s Depression Inventory (CDI) scores than those with heterozygous or compound heterozygous mutations (*p* = 0.041). Similarly, disease severity assessed using the ISSF was significantly higher among patients with homozygous *MEFV* mutations (*p* = 0.012). In contrast, Pittsburgh Sleep Quality Index (PSQI) scores did not differ significantly according to *MEFV* genotype (*p* = 0.288).

### 3.7. Correlation Analyses

**Figure 4:**
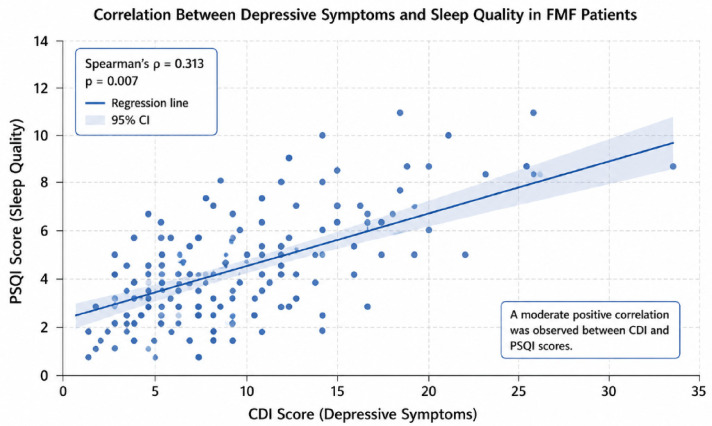
Correlation between Children’s Depression Inventory scores and Pittsburgh Sleep Quality Index scores in children and adolescents with Familial Mediterranean Fever.

Correlation analyses within the FMF cohort are summarized in Figure 4. A weak-to-moderate positive correlation was observed between PSQI and CDI scores (Spearman’s ρ = 0.313, *p* = 0.007). ISSF scores were not significantly correlated with either CDI or PSQI scores (both *p* > 0.05).

### 3.8. Multivariable Regression Analysis

Multivariable logistic regression analysis was performed to evaluate factors independently associated with poor sleep quality (PSQI ≥ 5). After adjustment for age and sex, FMF diagnosis was not independently associated with poor sleep quality (adjusted OR 0.61, 95% CI 0.31–1.18; *p* = 0.142). Increasing age was independently associated with poor sleep quality (adjusted OR 1.24, 95% CI 1.07–1.43; *p* = 0.003), whereas female sex was not independently associated with poor sleep quality (adjusted OR 1.18, 95% CI 0.62–2.27; *p* = 0.611). Detailed results of the multivariable logistic regression analysis are presented in Table 6.

## 4. Discussion

The present study evaluated sleep quality and depressive symptoms in a clinically stable cohort of children and adolescents with FMF receiving continuous colchicine therapy during attack-free periods. Four principal findings emerged. First, healthy controls demonstrated higher depressive symptom and sleep disturbance scores than patients with FMF. Second, sleep quality was significantly poorer among adolescents than younger children, independent of disease severity. Third, disease severity assessed using the ISSF was not associated with sleep quality or depressive symptoms. Finally, poorer sleep quality was moderately correlated with greater depressive symptom severity.

Previous studies have generally reported impaired health-related quality of life, increased depressive symptoms, anxiety, fatigue, and poorer sleep quality among children and adolescents with FMF compared with healthy peers [13,15,17,18,19,20,24,32]. These studies consistently indicate that psychosocial well-being may be adversely affected in pediatric FMF, particularly in relation to sleep quality, fatigue, and emotional functioning. The findings of the present study should therefore be interpreted as complementary rather than contradictory to the existing literature. Differences between previous studies and the present findings may partly reflect differences in study design and clinical context. Many previous studies included patients with varying levels of disease activity, evaluated participants during both attack and attack-free periods, or did not systematically assess disease severity using standardized instruments. In contrast, all patients in the present cohort were evaluated during clinically stable attack-free periods while receiving ongoing colchicine therapy, and disease severity was systematically assessed using the ISSF. This methodological approach minimized the potential influence of acute inflammatory episodes on sleep quality and emotional well-being and provided a more homogeneous clinical context for interpreting psychosocial outcomes.

One of the most notable findings of the present study was that healthy controls demonstrated significantly higher PSQI and CDI scores than children with FMF. Although this finding may initially appear counterintuitive, several factors should be considered when interpreting these results. First, the potential influence of acute inflammatory attacks, pain, fever, and disease-related sleep disruption was minimized by restricting assessments to clinically stable attack-free periods. Second, most patients demonstrated regular colchicine adherence and predominantly mild disease severity, indicating effective disease control within the study cohort. These clinical characteristics distinguish the present cohort from those included in many previous pediatric FMF studies, in which patients were evaluated across a broader spectrum of disease activity. Beyond disease control, regular clinical follow-up may also facilitate psychosocial adaptation through continuous family education, reassurance, and structured disease management. These findings should not be interpreted as indicating that FMF confers protection against sleep disturbances or depressive symptoms. Rather, they suggest that, under conditions of effective long-term disease control and regular multidisciplinary follow-up, psychosocial outcomes may approximate those observed in the general pediatric population, consistent with emerging resilience and family-centered adaptation frameworks in pediatric chronic disease [33,34].

Recent epidemiological studies have documented increasing rates of sleep disturbances, emotional distress, and depressive symptoms among children and adolescents, highlighting the importance of considering developmental and environmental influences when interpreting psychosocial outcomes in pediatric populations [35,36]. These influences may include academic demands, family environment, lifestyle factors, and broader societal changes that can affect psychological well-being independently of chronic disease status [35]. Accordingly, the higher psychosocial symptom scores observed among healthy controls in the present study should not necessarily be considered anomalous but may instead reflect contemporary trends in pediatric mental health. At the same time, growing evidence suggests that resilience, adaptive coping strategies, family support, and family-centered disease management may mitigate the psychosocial burden associated with chronic childhood diseases, indicating that emotional well-being is shaped not only by inflammatory burden but also by developmental, familial, and psychosocial factors [33,34]. Although these factors were not directly evaluated in the present study, they provide a plausible theoretical framework for interpreting why clinically stable children receiving regular long-term multidisciplinary care may demonstrate favorable psychosocial outcomes despite living with a chronic inflammatory disease. This interpretation should nevertheless be considered hypothesis-generating rather than confirmatory, given that resilience and family functioning were not directly assessed in the present cohort.

Adolescence is characterized by substantial biological, psychological, and social changes that directly influence sleep regulation. Circadian phase delay, increasing academic demands, digital media use, social stressors, and emotional vulnerability all contribute to impaired sleep quality during this developmental period [37]. Consistent with these observations, adolescents (13–18 years) in the present study demonstrated significantly higher PSQI scores and a markedly greater prevalence of poor sleep quality than younger children. In contrast, disease severity assessed using the ISSF did not differ significantly between age groups, suggesting that the observed age-related differences in sleep quality were unlikely to be explained by greater inflammatory disease burden alone. Furthermore, multivariable logistic regression demonstrated that increasing age remained independently associated with poor sleep quality after adjustment for sex and FMF diagnosis, further supporting the importance of developmental factors in sleep regulation. Taken together, these findings suggest that adolescence may represent a particularly vulnerable period for sleep disturbances in children with FMF, irrespective of clinically assessed disease severity.

No statistically significant association was identified between ISSF scores and psychosocial outcomes. However, this finding should be interpreted cautiously because the number of patients with severe disease was small, thereby limiting the statistical power of subgroup analyses. Notably, patients with severe disease demonstrated numerically higher CDI and PSQI scores and a greater frequency of poor sleep quality, although these differences did not reach statistical significance. Therefore, the absence of statistically significant associations should not be interpreted as evidence that disease severity has no influence on psychosocial functioning. Rather, the present findings indicate that, within this clinically stable cohort composed predominantly of children with mild-to-moderate disease receiving regular colchicine therapy, disease severity was not independently associated with sleep quality or depressive symptoms. Larger studies including a greater proportion of patients with severe disease are needed to further clarify the relationship between inflammatory disease burden and psychosocial outcomes. From a clinical perspective, these findings underscore the importance of routinely evaluating sleep quality and psychosocial well-being in adolescents with FMF regardless of disease severity.

Another important finding was a modest positive correlation between sleep quality and depressive symptoms, indicating that poorer sleep was associated with greater depressive symptom severity. This observation is consistent with the well-established bidirectional relationship between sleep regulation and emotional functioning in pediatric populations [22]. Previous studies have likewise demonstrated close associations among sleep disturbances, fatigue, emotional well-being, and health-related quality of life in children and adolescents with FMF [17,18,20,21]. Although the cross-sectional design precludes conclusions regarding causality, the observed association is biologically plausible and consistent with current evidence indicating reciprocal interactions between sleep and emotional health. Sleep disruption may impair emotional regulation and stress tolerance, whereas depressive symptoms may contribute to difficulties in sleep initiation and maintenance. In addition, inflammatory cytokines implicated in FMF pathophysiology, particularly interleukin-1β and tumor necrosis factor-α, have been shown to influence both sleep architecture and affective regulation through neuroimmune pathways [22,23]. These findings support the concept that sleep quality and emotional well-being should be considered closely interconnected domains when evaluating the overall health status of children and adolescents with FMF.

The present findings have several potential clinical implications. Even among clinically stable children and adolescents with FMF receiving maintenance colchicine therapy, sleep quality remained closely associated with emotional well-being, and adolescents appeared particularly vulnerable to sleep disturbances. These observations suggest that routine follow-up should extend beyond the assessment of inflammatory disease activity to include periodic evaluation of sleep and psychological well-being. Although no disease-specific intervention can be recommended based on the present findings, routine screening may facilitate early identification of children requiring additional psychosocial support. Integrating these assessments into multidisciplinary care may enable timely supportive interventions and ultimately contribute to improving health-related quality of life in pediatric patients with FMF.

The present study has several strengths. To our knowledge, it is among the few pediatric FMF studies to evaluate sleep quality and depressive symptoms exclusively during clinically stable attack-free periods while simultaneously assessing disease severity using the ISSF. By including only patients receiving maintenance colchicine therapy and evaluating them during routine attack-free follow-up, the study minimized the potential confounding effects of acute inflammatory activity on patient-reported outcomes. In addition, age-stratified analyses and multivariable logistic regression provided a more robust assessment of the influence of developmental factors on sleep quality.

Several limitations should also be acknowledged. First, the cross-sectional design precludes causal inference regarding the relationships among sleep quality, depressive symptoms, and disease severity. Second, the study was conducted at a single tertiary care center with a relatively modest sample size, particularly in the severe disease subgroup, which may have limited statistical power to detect associations with disease severity. Accordingly, the absence of statistically significant associations with disease severity should be interpreted with caution. Third, psychosocial variables including screen time, academic stress, socioeconomic status, physical activity, sleep hygiene, family functioning, and resilience were not systematically assessed because these variables were not included in the original study protocol. Finally, although the PSQI has demonstrated acceptable validity in school-aged children and adolescents [30,31], younger participants occasionally required investigator clarification of questionnaire items, which may have influenced self-reported responses despite efforts to avoid interviewer bias. Future prospective multicenter studies incorporating comprehensive psychosocial, family, and resilience-related assessments are warranted to clarify the complex interactions among inflammatory activity, developmental factors, family environment, sleep quality, and emotional well-being in pediatric FMF.

## 5. Conclusions

In conclusion, the present findings suggest that overall well-being in children and adolescents with FMF may be influenced not only by inflammatory disease characteristics but also by developmental and family-related factors. Within this clinically stable cohort receiving maintenance colchicine therapy, age appeared to be more closely associated with sleep quality than clinically assessed disease severity, whereas poorer sleep quality was moderately associated with greater depressive symptom severity. Collectively, these findings support a comprehensive biopsychosocial approach to the long-term care of pediatric FMF. Future prospective multicenter studies incorporating detailed assessments of psychosocial functioning, family environment, and resilience are warranted to further clarify the complex interactions among inflammation, development, sleep, and emotional well-being throughout childhood and adolescence.

## Figures and Tables

**Table 1:** Summary of previous studies evaluating psychosocial outcomes in pediatric Familial Mediterranean Fever.

Study	Population	Assessment Tools	Main Findings	Attack-Free Assessment	Standardized Disease Severity Assessment
İncesu et al. **[17]**	Children and adolescents with FMF and healthy controls	PSQI, PedsQL-MFS, ISSF	Poorer sleep quality and greater fatigue were observed in FMF patients. Sleep quality and fatigue correlated with disease activity, attack frequency, and ISSF scores, whereas *MEFV* mutation status was not associated with these outcomes. Adolescents showed poorer sleep quality than younger children	No	Yes (ISSF)
Durcan et al. **[18]**	Children and adolescents with FMF	PSQI, anxiety/depression scales	Sleep disturbances were associated with anxiety and depressive symptoms	Partially	No
Makay et al. **[19]**	Pediatric FMF patients	Pittsburgh Sleep Quality Index (PSQI)	Poor sleep quality was more frequent in children with FMF than in healthy controls	No	No
Küçükşahin et al. **[20]**	Pediatric FMF cohort	Sleep quality scales	Poor sleep quality was associated with higher disease activity	No	Limited (non-standardized assessment)
Saraç et al. **[21]**	Adolescents with FMF	PedsQL Multidimensional Fatigue Scale, Sleep Quality Scale	Fatigue was associated with recent attacks, shorter sleep duration, lower physical activity, and poorer sleep quality; no association with the M694V mutation was observed	No	No
Makay et al. **[24]**	Pediatric FMF patients	Anxiety and depression scales	Higher anxiety and depressive symptom scores compared with healthy controls	No	No
Present study	Clinically stable pediatric FMF patients	CDI, PSQI, ISSF	Sleep quality and depressive symptoms were evaluated during clinically stable attack-free periods. Associations with standardized disease severity and developmental stage (childhood vs. adolescence) were investigated	Yes	Yes (ISSF)

**Table 2:** Sociodemographic and clinical characteristics of children and adolescents with Familial Mediterranean Fever and healthy controls.

Characteristic	FMF (*n* = 72)	Controls (*n* = 88)	*p* Value
Age (years), mean ± SD	10.6 ± 3.3	11.5 ± 3.1	0.081
Female sex, n (%)	35 (48.6)	44 (50.0)	0.861
**FMF-specific characteristics**			
Age at symptom onset (years), mean ± SD	6.2 ± 3.3	—	—
Age at diagnosis (years), mean ± SD	7.9 ± 3.4	—	—
Age at colchicine initiation (years), mean ± SD	7.9 ± 3.4	—	—
Regular colchicine adherence, n (%)	62 (86.1)	—	—
Irregular colchicine adherence, n (%)	10 (13.9)	—	—
Homozygous *MEFV* mutation, n (%)	18 (25.0)	—	—
Compound heterozygous ***MEFV*** mutation, n (%)	31 (43.1)	—	—
Heterozygous ***MEFV*** mutation, n (%)	23 (31.9)	—	—

**Footnote:** Data are presented as mean ± standard deviation (SD) or number (percentage) unless otherwise indicated. *p* values compare the FMF and control groups. FMF-specific clinical characteristics were evaluated only in patients with FMF. **Abbreviations: FMF**, Familial Mediterranean Fever; ***MEFV***, Mediterranean fever (*MEFV*) gene; **SD**, standard deviation.

**Table 3:** Comparison of psychosocial assessment outcomes between children and adolescents with Familial Mediterranean Fever and healthy controls.

Variable	FMF (*n* = 72)	Controls (*n* = 88)	*p* Value
**CDI score, mean ± SD**	8.2 ± 5.3	10.3 ± 6.8	0.048
**PSQI score, mean ± SD**	3.0 ± 2.3	4.7 ± 2.7	<0.001
**Poor sleep quality (PSQI ≥ 5), n (%)**	18 (25.0)	41 (46.6)	0.006
**Clinically significant depressive symptoms (CDI ≥ 19), *n* (%)**	4 (5.6)	8 (9.1)	0.411

**Footnote:** Data are presented as **mean ± standard deviation (SD)** or **number (percentage)** unless otherwise indicated. *p* values compare the FMF and control groups. The ISSF score was assessed only in participants with FMF. **Abbreviations: CDI**, Children’s Depression Inventory; **FMF**, Familial Mediterranean Fever; **ISSF**, International Severity Scoring System for Familial Mediterranean Fever; **PSQI**, Pittsburgh Sleep Quality Index; **SD**, standard deviation.

**Table 4:** Psychosocial assessment findings according to disease severity assessed using the International Severity Scoring System for Familial Mediterranean Fever.

ISSF Category	*n* (%)	CDI Score, Mean ± SD	PSQI Score, Mean ± SD	Poor Sleep Quality (PSQI ≥ 5), *n* (%)
**Mild (0–2)**	39 (54.2)	7.5 ± 4.9	2.8 ± 2.1	10 (25.6)
**Moderate (3–5)**	28 (38.9)	8.9 ± 5.4	3.2 ± 2.4	5 (17.9)
**Severe (≥6)**	5 (6.9)	11.6 ± 6.1	5.2 ± 2.4	3 (60.0)
***p*** **-value**	—	0.182	0.146	0.071

**Table 5:** Age-stratified comparison of psychosocial outcomes among children and adolescents with Familial Mediterranean Fever.

Variable	6–12 Years (*n* = 51)	13–18 Years (*n* = 21)	*p* Value
**CDI score, mean ± SD**	7.9 ± 5.1	8.9 ± 5.7	0.462
**PSQI score, mean ± SD**	2.5 ± 2.0	4.2 ± 2.7	0.005
**ISSF score, mean ± SD**	2.3 ± 1.1	2.6 ± 1.3	0.334
**Poor sleep quality (PSQI ≥ 5), *n* (%)**	7 (13.7)	11 (52.4)	0.005

**Footnote:** Data are presented as mean ± standard deviation (SD) or number (percentage). *p* values were calculated using the Mann–Whitney U test for continuous variables and the chi-square test or Fisher’s exact test, as appropriate. **Abbreviations: CDI**, Children’s Depression Inventory; **FMF**, Familial Mediterranean Fever; **ISSF**, International Severity Scoring System for Familial Mediterranean Fever; **PSQI**, Pittsburgh Sleep Quality Index; **SD**, standard deviation.

**Table 6:** Multivariable logistic regression analysis of factors independently associated with poor sleep quality (PSQI ≥ 5).

Variable	Adjusted Odds Ratio (OR)	95% Confidence Interval	*p* Value
**FMF diagnosis**	0.61	0.31–1.18	0.142
**Age (per 1-year increase)**	1.24	1.07–1.43	0.003
**Female sex**	1.18	0.62–2.27	0.611

**Footnote:** Multivariable logistic regression analysis was performed with poor sleep quality (PSQI ≥ 5) as the dependent variable. Odds ratios (ORs) are adjusted for age, sex, and FMF diagnosis. A *p*-value < 0.05 was considered statistically significant.

## Data Availability

The data presented in this study are available on reasonable request from the corresponding author due to ethical restrictions involving pediatric participants and the need to protect participant privacy and confidentiality.

## Supplementary Materials

The following supporting information can be downloaded at: https://www.mdpi.com/article/10.3390/children13070950/s1. Table S1: Genotype-Based Comparison of Psychosocial Outcomes in FMF Patients.

## Abbreviations

CI	Confidence Interval
FMF	Familial Mediterranean Fever
OR	Odds Ratio
PSQI	Pittsburgh Sleep Quality Index
