# Supplementary material for: Sleep Quality and Depressive Symptoms in Clinically Stable Pediatric Familial Mediterranean Fever: Associations with Disease Severity and Developmental Factors
Source: Children (Basel). 2026 Jul 20;13(7):950. doi: 10.3390/children13070950 (PMC13406458; doi:10.3390/children13070950)
Supplement: Supplementary file 1 [file children-13-00950-s001.zip › children-4336957-supplementary.pdf]

Supplementary Table S1. Genotype-Based Comparison of Psychosocial Outcomes in FMF Patients

Variable	Homozygous (n=18)	Compound heterozygous (n=31)	Heterozygous (n=23)	p-value
**CDI score**	10.2 ± 5.8	8.1 ± 5.2	6.9 ± 4.7	0.041
**PSQI score**	3.6 ± 2.5	3.0 ± 2.3	2.7 ± 2.0	0.288
**ISSF score**	3.1 ± 1.4	2.3 ± 1.1	1.9 ± 0.9	0.012

Footnote: Comparisons between genotype groups were performed using the Kruskal–Wallis test. Data are presented as mean ± standard deviation (SD). CDI: Children's Depression Inventory; ISSF: International Severity Scoring System for FMF; PSQI: Pittsburgh Sleep Quality Index.
